# Epidemiology of *Clostridium Difficile* Infection in a Large Hospital in Northern Italy: Questioning the Ward-Based Transmission

**DOI:** 10.2174/1874285801711010360

**Published:** 2017-12-29

**Authors:** Gabriella Piatti, Marco Bruzzone, Vincenzo Fontana, Alessandro Mannini, Marcello Ceppi

**Affiliations:** 1 DISC, Department of Surgical Sciences and Integrated Diagnostics, University of Genoa, Genoa, Italy; 2 Division of Microbiology, Ospedale Policlinico San Martino, Genoa, 10 Largo Benzi, 16132, Genoa, Italy; 3 Unit of Clinical Epidemiology, Ospedale Policlinico San Martino, Genoa, 10 Largo Benzi, 16132, Genoa, Italy; 4 Department of Science, Environment and Life, University of Genoa, Genoa, Italy

**Keywords:** *Clostridium Difficile* Infection, Diarrhea, Statistic, Human, Contagion, Hospital, Ecology

## Abstract

**Background:**

*Clostridium Difficile* infection (CDI) is considered a ward-based nosocomial infection, due to contagion among patients. Molecular studies recently questioned ward-based contact for disease spread.

**Objective:**

To investigate whether it is plausible that CDI spread in San Martino Hospital of Genoa was due to a ward-based contact and patient-to-patient diffusion.

**Methods:**

We conducted a retrospective cohort study of CDI cases from April 2010 to March 2015. We referred to Hospital data set and Admission Service. Multilevel modelling approach and ecological analysis were used to assess *C. difficile* infection risk according to wards and time of occurrence. Six representative CD strains were ribotyped to assess a possible equivalence.

**Results:**

The assessment of 514 CDI cases showed that the risk of disease and rate of incidence in wards were independent, while frequency of cases and number of wards involved exhibited a positive relationship, excluding the typical epidemic pattern of contagious diffusion, *i.e*., many cases in few wards. The extra-binomial variability due to ward clustering was not significant, indicating homogeneity in the probability of CDI occurrence across all wards. Three hundred sixty-eight patients changed ward, without showing connection between the frequency of cases in new wards and incidence among new subjects. Trigonometric components described a significant contribution of seasonality, with excess of CDI cases during the winter months. Molecular analysis showed different ribotypes of CD strains from the same ward.

**Conclusion:**

From our results it seems unlikely that in our institution CDI occurrence is due to ward-based contact and inter-human contagion of the organism.

## 1 INTRODUCTION

*Clostridium Difficile* (CD) is an anaerobic spore forming bacterium, responsible for CD infection (CDI), an acute disease of human bowel, which can cause varying levels of diarrhea, pseudomembranous colitis and lethal toxic mega colon [1]. CDI is the most frequent cause of nosocomial diarrhea, also known as a hospital-acquired infection due to spread of the microorganism among subjects lying in hospital settings [2]. The whole comprehension of CDI pathogenesis is complicated by hurdles such as the possible presence, in the human bowel, of vegetative and spore bacterial forms, the latter responsible for asymptomatic human carriers and recurrent episodes of disease [3]. The severe limits of current diagnostic tools, which lack in sensitivity and/or specificity, also hinder precise evaluations of the real number of CD carriers and affected people, both in the hospital environment and in the community [4]. Crucial aspects and important queries do emerge from recent works in the field. CDI continues to be a growing health care problem, also in those wards where preventive strategies, such as isolation of affected subjects and hygienic measures, based on assumption of patient-to-patient contagion, are applied [5]. Studies based on Polymerase Chain Reaction (PCR) ribotyping of CD isolates clearly showed that genomic identity among the strains is often absent [6]. The advent of genome micro-evolutionary analysis and whole-genome sequencing allowed further demonstration that fewer cases of CDI than expected by using PCR ribotyping could be attributed to person-to-person CD contagion in hospital environments [7, 8]. In those investigations, it emerges that hospital-acquired episodes account for the minority of the hospital-associated cases considered. Epidemiological analyses could similarly be useful to exclude direct transmission of CDI among patients, and transmission between the environment and subjects themselves, when individual cases occur in different wards [6].

We performed a retrospective spatio-temporal analysis and field epidemiology investigation of CD toxin B (CDTB) analyses performed in hospital during a period of 60 months, and we selected six representative CD strains for ribotyping. Our aim was to evaluate whether patient-to-patient transmission of CD can be convincing or whether alternative hypotheses and preventive strategies may be considered. Among these is an interesting hypothesis proposing CD as a foodborne bacterium [9]. Nowadays, this possibility is rarely addressed as a source of infection, although CD isolates from various types of foods, animals and vegetables, show common genotypes with isolates from humans [9-13].

## 2 MATERIALS AND METHODS

### 2.1 Study Population

The source cohort consisted of patients affected by diarrhea, admitted to the San Martino Hospital in Genoa, Italy, grouped by ward, and subjects in the community from the same regional area, whose stools were sent to the microbiology laboratory for CDTB detection. In the hospital, the spatial unit was the ward, which is a unit provided with rooms where a unique staff of health-care and co-workers are active. The study period was from April 2010 to March 2015. We evaluated the San Martino Hospital data set (TD-Synergy MultiLab, SIEMENS) including all CDTB analyses performed. The person-days of hospitalization were obtained for groups of wards through the Hospital Admission Service, Business Intelligence Discoverer Desktop (Oracle).

The Institutional Ethics Committee of San Martino University Hospital approved the study (n. reg. CEA 13/11-Progetto istMicro1/2011), by dispending from informed consents.

### 2.2 Definition of Outcome

CDI cases were defined as subject with at least one positive stool for CDTB. Toxin B was detected in fecal specimens through the Rapid Membrane Enzyme Immunoassay (Techlab Inc.) according to the manufacturer's instructions. We considered the total number of patients and analyses, including the analyses repeated for single subjects and changes of result concerning single patients during time. We also reviewed the hospital database and the movements of patients analyzed for CDTB throughout the hospital, from the hospital to the community and vice-versa.

### 2.3 Molecular typing of *C. Difficile* Strains

Six CD strains, isolated from CDTB positive stools from six patients, were analyzed in order to identify the ribotypes. Five strains were isolated during a period spanning eighteen days, between December 2012 and January 2013. Among these, three strains were isolated from three cases observed in three different wards, while two were from two cases admitted in the same ward. The sixth strain was isolated outside the above-mentioned group, *i.e*., later and from another ward yet.

For the molecular analyses, fresh fecal specimens were grown in anaerobiosis at 37^o^C for 48 hours on *Clostridium difficile* CDMN agar (MEUS, Italy). DNA was extracted from colonies and PCR ribotyping was performed at the Istituto Superiore di Sanità (ISS) in Rome, according to the method described by Bidet [18].

### 2.4 Statistical Analyses

The first step in data analysis was to check whether in wards where two or more CDTB positive stools occurred, *i.e*., wards with an apparent epidemic risk, the proportion of positive samples was higher than in wards with only one occurrence. Wards with the same numbers of CDTB positivity were aggregated and the odds ratio (OR), along with the corresponding 95% confidence interval (95% CI), were computed to compare the proportions [14]. An OR > 1 suggested a higher likelihood of positive analyses in wards with epidemic risk. The opposite occurred with an OR < 1. Moreover, if the 95% CI did not include the value 1, the probability that compared proportions would not differ was < 5%.

Our data showed two levels of clustering, *i.e*., possible different wards of admission for a single patient, and the possible occurrence of multiple CDTB analyses for the same patient. Therefore, multilevel mixed effect logistic regression modelling was performed considering the proportion of positive analyses as dependent variables to estimate the OR [15]. Furthermore, since various patients were hospitalized in more than one ward, the clusters were treated as cross-classified [15].

This model also allowed us to assess whether any characteristics of patients could predict the occurrence of CDTB positivity, namely, age, gender, calendar year of analysis performance, patients’ origin (nosocomial or community) and seasonality (trigonometric function with a 3-month period), checking for extra-variability in the dependent variable due to the hierarchical, *i.e.*, clustered, data nature. Accordingly, ORs of wards with two or more CDTB positivity were adjusted for potential confounding due to these characteristics. The same procedure was subsequently applied only in the six months in which the highest absolute number of CDTB positivity occurred, *i.e*., more than 90th percentile of the monthly distribution.

The proportion of patients who became CDTB positive after admittance to a new ward was linked to the number of CDTB cases occurring in that ward by means of the logistic model. A similar model was employed to assess whether the frequency of cases observed in each ward was related to the admission of patients before positivity in other wards. In this case, the model was applied to a count, *i.e*., the number of CDTB cases observed in the ward. We thus used the Poisson regression model instead of the logistic model.

Lastly, using person-days of hospitalization, crude incidence rates (CIR) of CDTB positivity were computed for an ecological analysis, combining wards with the same number of cases. The expected number of events for each ward was also calculated, assuming the rate of positive CDTB incidence of the entire hospital as the reference. Statistical analyses were performed with STATA and MLWin statistical software [16, 17].

## 3 RESULTS

During the 60-month period, 4590 stool samples were analyzed for detection of CDTB presence. Samples came from 3178 subjects, inpatients hospitalized in different wards and outpatients from the same regional area. Over the period, CDTB was identified in 557 specimens from 514 patients, 472 inpatients and 42 outpatients (Table **1**).

### 3.1 Distribution of CDTB Positive Analyses by Place and Time

**Fig. (1):**
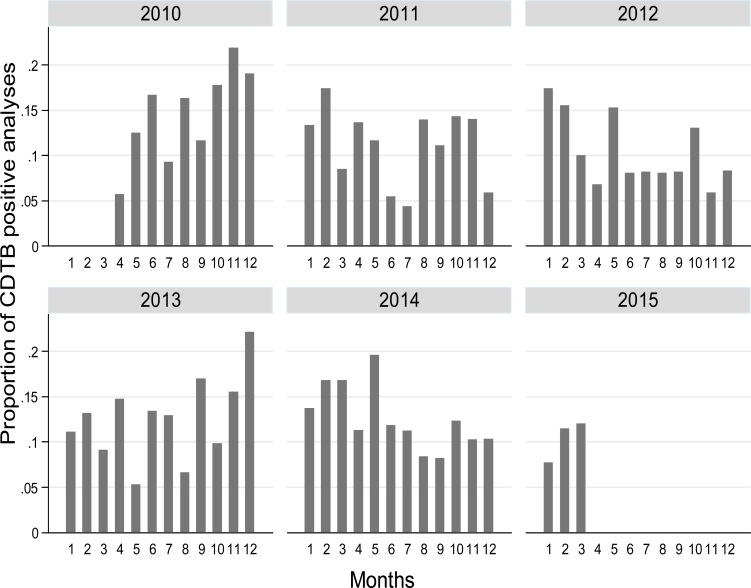
Distribution of proportion of CDTB positive analyses by month and year, from April 1, 2010, to March 31, 2015 in San Martino Hospital, Italy.

The distribution of the proportion of CDTB positive analyses by month and year shows a clear pattern of increased test positivity during colder periods (Fig. **1**).

We grouped the wards according to the number of positive analyses and considered the proportion of positive tests and proportion rank, compared to the frequency of CDTB positive analyses. The proportion of positive analyses does not appear to increase as much as the number of CDI cases; in fact, the proportion for 1 case (12.4) has a rank of 10 out of 18 (Table **2**).

**Fig. (2):**
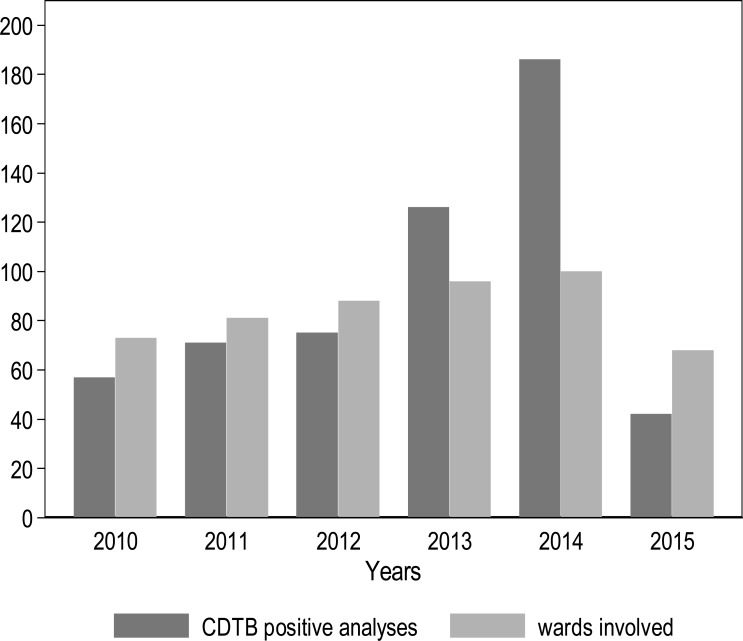
Frequency of positive CDTB analyses and number of wards of San Martino Hospital, Italy, involved by year, from April 1, 2010, to March 31, 2015.

The evaluation of the number of wards involved in cases and the frequency of positive CDTB analyses over the years demonstrate that the two items exhibit a positive relationship (Kendall tau=1; *p*-value=0.009) (Fig. **2**), while typical epidemic patterns are characterized by many cases gathered in few wards.

**Fig. (3):**
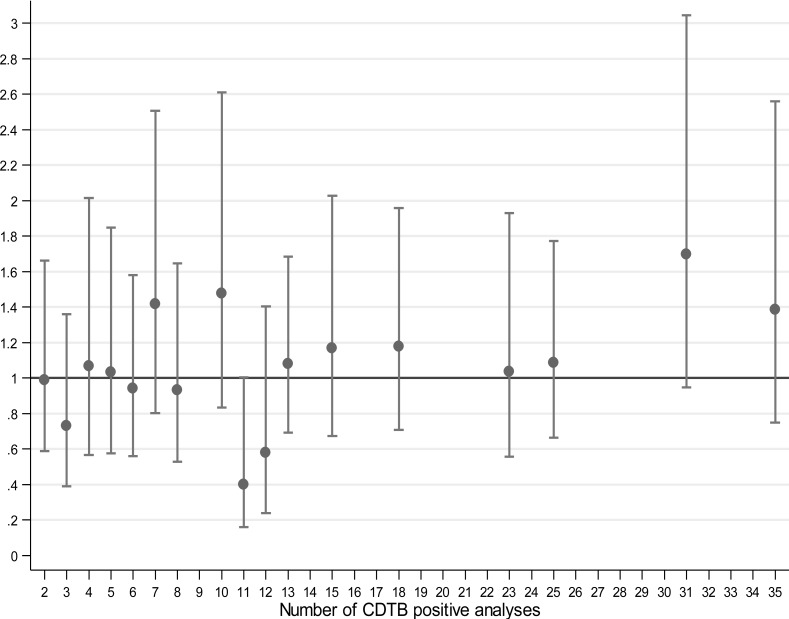
Multilevel logistic modeling of risk of CDTB positivity in wards with positive CDTB > 1 compared to wards with a single positivity in San Martino Hospital, Italy.

The risk of positivity in wards with two or more CDTB cases was compared to those with a single positive analysis from the multilevel logistic model (Fig. **3**).

Very few wards with 31 positive analyses resulted higher giving OR > 1 (OR = 1.70; 95% CI 0.95-3.04), while most wards gave rise to OR values very close to one, suggesting independence between the observed positive CDTB results and frequency of the same. This model was applied to the six months with the highest absolute frequency of CDTB positive analyses, *i.e*., Dec 2013, Jan, Feb, Mar, May and Oct 2014, for 603 analyses including 123 with positive result (Table **3**). Although the ORs do not show an upward trend and are not parallel to the increase in number of positive analyses, all OR values are greater than one, suggesting that excess of cases may really occur in wards with a high frequency of disease in particular periods (“at-risk months”).

### 3.2 Distribution of CDTB Positive Analyses by Place and Time According to Patients Movements

During the period of observation, 368 subjects changed ward of admission within the hospital, giving 461 movements. Fifty-five subjects became CDTB positive in new wards, without any link between frequency of turning positive and frequency of cases observed in new wards (*p* = 0.420). Not even the arrival of sixty-three patients, positive in previous wards, was related to the number of positive analyses observed in the new settings (*p* = 0.975) (Data not shown). This analysis confirms what has already been demonstrated, *i.e*., the lack of risk of CDI related to the wards.

### 3.3 CDI Risk According to Patients’ Characteristics

When the multilevel logistic model concerns characteristics of positive CDTB patients (Table **4**), it is noteworthy that the level of extra-binomial variability due to ward clustering is not important (*p* = 0.610). This finding indicates a substantial homogeneity in the probability of being CDTB positive across all wards considered in the analysis.

**Fig. (4):**
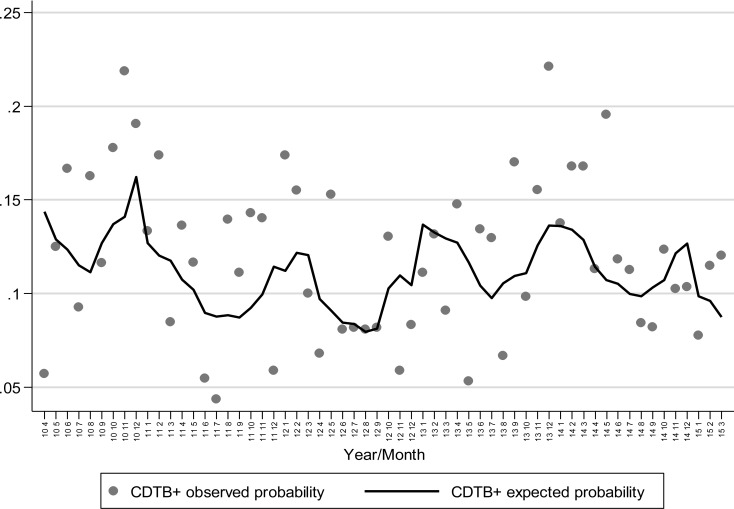
Observed proportion of CDTB positivity and probability of positivity expected plotted against months/years.

Patient’s origin has no remarkable influence on the probability of achieving positive results in CDTB detection, while advanced age increases the risk of getting CDI. Trigonometric components (sine and cosine) confirm the contribution of seasonality to CDI onset shown in Fig. (**1**), (*p* = 0.029), also depicted when the observed proportion of positivity and the probability of positivity expected from the logistic model are plotted against months-years (Fig. **4**). Here, the statistical modelling highlights that excess of positive CDTB analyses are to be expected during winter months, separated by reductions in the hottest seasons.

### 3.4 Ecological Analysis

**Fig. (5):**
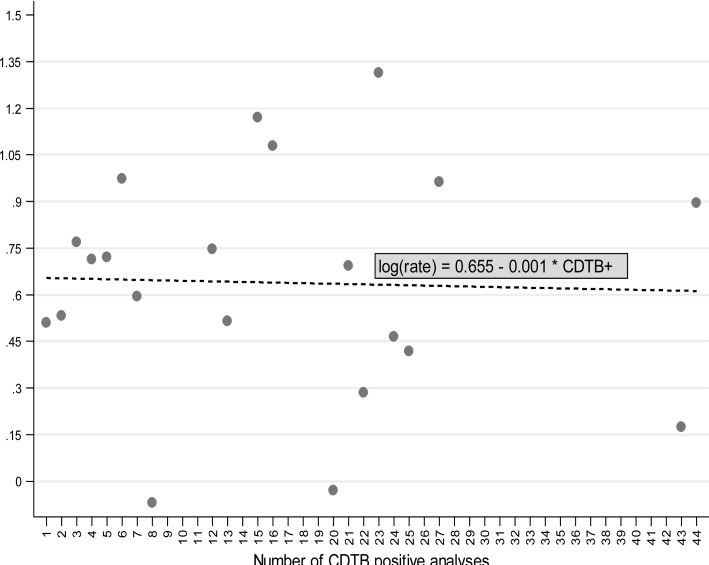
Relationship between the log-transformed CDTB CIR (crude incidence rates), resulting from the evaluation of person-days of hospitalization, and the number of positive CDTB analyses (slope = -0.001; p = 0.89).

The use of crude incidence rates, resulting from the evaluation of person-days of hospitalization, allowed consideration of the connection between the log-transformed crude incidence rates of CDTB positivity and the number of events (Fig. **5**). Increasing the positive analysis, log CIR declines slightly (slope = -0.001; *p* = 0.89). In practice, this analysis also stressed the substantial independence between the probability of being CDTB positive and the number of observed positive results, previously demonstrated with different evaluations (Tables **2**, **3**; Figs. **2**, **3**).

**Fig. (6):**
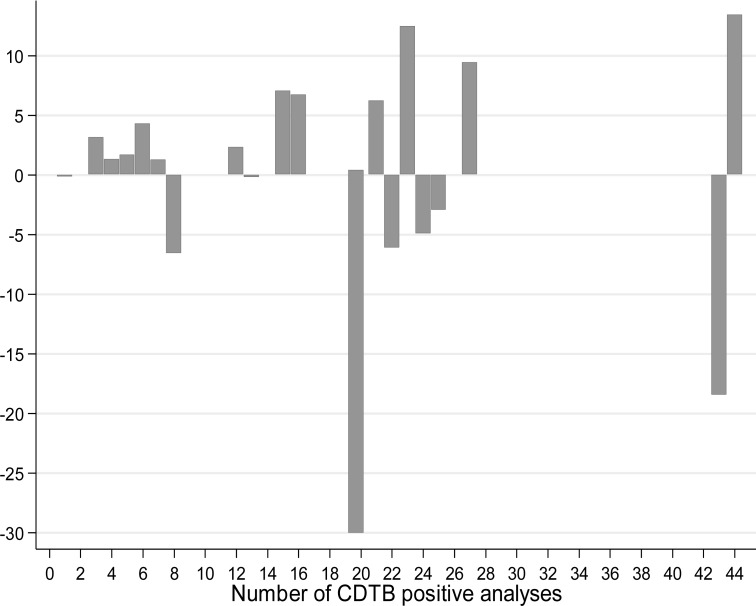
Plotting of difference between observed and expected CDTB positivity by combining wards with equal numbers of events.

Finally, we combined the wards with an equal number of cases to draw the difference between observed and expected CD cases (Fig. **6**).

Also this graph allows it to be noted that, in the case of a homogeneous spatial distribution of CDI incidence, the lack of cases is also present in areas where large numbers of events have occurred anyway.

### 3.5 Molecular Typing of *C. Difficile* Strains

Molecular analysis of few but representative CD strains supports, in a small size, the results obtained with the epidemiological investigation. Within the small group of five strains isolated in the same time period, the two coming from the same ward and eight days apart, belonged to different ribotypes, *i.e*., 018 and 607 (Data not shown). In this case, the different ribotype excluded a person-to-person transmission. Within the same group, three strains, each of which came from three different wards, belonged to the same type 607. In this case, the inter-human transmission could be excluded through the different provenance, despite belonging to same ribotype, the same of the sixth strain, coming from another ward still and in a different time period.

## 4 DISCUSSION

The cross-classified multilevel logistic model, concerning individual data, enabled us to assess the risk of CDI compared to the incidence of cases that occurred in single wards. It also allowed us to evaluate the trend of the disease over time and the influence of individual patient characteristics on the disease incidence. The ecological approach employed the frequency of hospital admissions to estimate the crude incidence rate of the disease.

Overall, these analyses indicate that 1) The risk of disease, and/or the rate of incidence, does not increase with increasing frequency of CDIs and its heterogeneity among different wards is not important; 2) the incidence of CDI does not correlate with the origin of patients, nosocomial or community, but with advanced age; 3) CDIs occur with some seasonality, *i.e*., more frequently in the winter, when peak numbers of cases occur and 4) an excess of CDI cases may truly occur in wards with high frequencies of disease only in particular periods (“at-risk months”).

One of the new discoveries of this work shows that CDI does not prevail in hospitals rather than outside in the community, as suggested in other studies [2]. We obtained our results through the analysis of nosocomial and true community cases, *i.e.,* from ambulatory centers, within the same regional area, differently from guidelines, that classify health-care facility associated (HCFA) CDI cases as community onset (CO) or community associated (CA) [19]. Another important discovery shows that the occurrence of nosocomial disease is not linked to the hospital environment, as previously suggested [20]. The connection between CDI onset and advanced age, and the seasonal variations characterized by higher incidence of CDI in the winter, show that the basic data in this investigation do not differ from previous studies [2, 21, 22].

In our epidemiological study, the length of the observational period was enough to observe a lack of connection between CDIs and wards, disproving ward-based transmission of CD and inter-human contagion. We took into account the risk of disease and its heterogeneity as crucial parameters for infections when related to the location of the events. In fact, when substantial, the heterogeneity would support the hypothesis of a spatial component of the disease and the presence of epidemic areas [14], which can demonstrate infection contagion from one person to another. Both of the approaches we utilized seem to agree that CDI risk and concentration of cases were not strictly connected, the risk was substantially homogenous across all wards and, except for a few wards characterized by a greater number of events, the occurrence of epidemics in single wards was only illusory. We also found a positive relationship between the frequency of cases and the number of wards involved, further contradicting the occurrence of ward-based contact for diffusion of CDI.

The asymptomatic presence of CD in spore form in the human bowel and the variable process of germination make it difficult to locate the source of the microorganism [1, 3]. Furthermore, different studies report contrasting explanations about the dynamics of CDI germination and the related risks [23, 24]. Thus, the lack of a real understanding of the pathogenesis of infection clouds the knowledge of its real incidence in different contexts and places. Most epidemiological studies on CDI, which suggest that the infection prevails in a hospital setting, also suggest that the microorganism spreads through ward-based contact, directly between patients or through medical staff, and from the environment [20]. Several genomic investigations contradicted the possibility of ward-based contact for many cases, having shown that CD strains isolated from different patients hospitalized in common wards belonged to different types [7]. Based on epidemiological linkages, health-care workers are considered the infective links for bacterial transmission among patients [25, 26]. A very low risk of CDI among household contacts and the absence of outbreaks in the related families contradict the responsibility of health-care workers in diffusion of the disease [27]. In the same manner, a representative ribotyping and the epidemiological-statistical analysis we performed allowed exclusion of the possibility that CDI diffusion in our institution occurred through patient-to-patient contagion.

Genomic analyses, spatio-temporal and field epidemiological investigations can be misleading since they are not suitable for discovering coincidences in the case of spatial connections of infectious events or genomic similarities between bacterial strains. Only the exclusion of person-to person contagion, by excluding the sharing of common spaces between different cases occurred in the same period, or by excluding genomic equivalence between strains isolated in the same period, even if in a common space, is indisputable and can leave room for plausible hypotheses.

In addition to excluding ward-based contact and inter-human contagion for CDI in the San Martino Hospital, this study also reports a relevant number of CDI cases that occurred in wards with high frequencies of disease just in particular periods. This finding suggests the existence of a single, periodic and common source of the infectious agent, which could be food.

Recent studies have begun to consider food as an important source of community-acquired infection. However, the same investigations consider person-to-person or surface-to-person transmission of CD as important in hospital, while they contemplate CD foodborne transmission along with other routes, such as zoonotic, waterborne, environmental and person-to-person [28, 29]. Therefore, a univocal and acceptable explanation for CD diffusion is still absent, while the mechanistic microbiological rationale indicates that ecology, rules and pathways for diffusive infectious diseases are unique and specific [9]. In the context of inter-human communicable diseases, the strictly human microorganisms, such as *Salmonella typhi* and *Shigella dysenteriae*, are enteric pathogens whose transmission in developed countries was broken by separating clean and polluted water [30]. Organisms that are not strictly human pathogens, such as non-typhoidal Salmonellae, Listeria and Yersinia, are routinely detected in food. They can be responsible for infective episodes caused by products that escape routine control procedures, and are confined to the subjects who ingest them. *C. difficile*, like other spore forming bacteria, is an environmental not strictly human bacterium, which can reach the human and animal bowel, presumably through food, which is not routinely investigated for presence of clostridia [30, 31].

## CONCLUSION

In conclusion, the epidemiological approach that we utilized has produced data corresponding with the basic rules of microbiological rationale. CD should be considered a foodborne pathogen not transmittable among people.

## Figures and Tables

**Table 1:** Demographic characteristics of hospitalized inpatients and outpatients with stools analyzed for *C. difficile* toxin B detection.

	**CDTB Analyses**		**Total** N (%°)
**Patients Variables**	**Negative** N (%*)	**Positive** N (%*)	
**Gender**			
Females	2,164 (86.6)	335 (13.4)	2,499 (54.4)
Males	1,869 (89.4)	222 (10.6)	2,091 (45.6)
**Age, years**			
0-64	1,270 (91.5)	118 (8.5)	1,388 (30.2)
65-79	1,344 (88.2)	180 (11.8)	1,524 (33.2)
>=80	1,419 (84.6)	259 (15.4)	1,678 (36.6)
** Patients provenance**			
hospital	3,622 (87.6)	511 (12.4)	4,133 (90.0)
community	411 (89.9)	46 (10.1)	457 (10.0)
**Year of analysis**			
2010 ^a^	341 (85.7)	57 (14.3)	398 (8.7)
2011	558 (88.7)	71 (11.3)	629 (13.7)
2012	623 (89.3)	75 (10.7)	698 (15.2)
2013	846 (87.0)	126 (13.0)	972 (21.2)
2014	1,297 (87.5)	186 (12.5)	1,483 (32.3)
2015^ b^	368 (89.8)	42 (10.2)	410 (8.9)
**Season**			
Winter	1,212 (87.1)	179 (12.9)	1391 (30.3)
Spring	960 (87.8)	133 (12.2)	1,093 (23.8)
Summer	855 (89.5)	100 (10.5)	955 (20.8)
Autumn	1,006 (87.4)	145 (12.6)	1,151 (25.1)

^*^ Row percentage.

^°^Column percentage.

^a^9 months.

^b^3 months.

**Table 2:** Proportion of CDTB positive analyses and rank by wards group.

**Number of** **Positive Analyses**	**Number of** **Wards**	Ward Groups*	**Total Number of** **Positive Analyses**	**Number of** **Performed Analyses**	**Proportion of** **Positive Analyses**	**Rank of** **Proportion**
1	35	1,3,4,6,10,12,13,15,16,17,18	35	280	12.4	10
2	17	1,4,6,9,12,15,16,17,20	34	250	13.6	13
3	6	2,3,5,6,10,12	18	214	8.4	2
4	4	10,12,20	16	132	12.1	8
5	5	7,10,15,16	25	214	11.7	5
6	6	1,3,10,15,17,19	36	305	11.8	6
7	4	4,7,10,17	28	177	15.8	15
8	3	5,7,15	24	232	10.3	3
10	3	3,15	30	189	15.9	16
11	1	1	11	146	7.5	1
12	1	1	12	102	11.9	7
13	5	4,7,8,10,19	65	586	11.1	4
15	2	8,10	30	242	12.4	9
18	3	10,15	54	370	14.6	14
23	1	10	23	184	12.5	11
25	2	10,15	50	374	13.4	12
31	1	10	31	153	20.3	18
35	1	10	35	198	17.7	17

1) Ambulatory Care; 2) Cardiac Surgery; 3) Emergency Room, Emergency Surgery, Observation; 4) Functional and Neurological Rehabilitation; 5) Gastroenterology; 6) General-Abdominal-Oncological-Thoracic- Surgery-Urology; 7) Hematology-Bone Marrow Transplantation Centre; 8) Infectious Diseases ; 9) Specialist Surgeries; 10) Medicine-Medicine intermediate Care; 11) Neonatology-New-Born Diseases; 12) Neurology-Ictus Centre Intensive Therapy-Psychiatry; 13) Neurosurgery-Neurosurgery for Trauma; 14) Obstetrics and Gynecology; 15) Oncology and Palliative Care; 16) Reanimation-Intensive Care- Sub intensive Therapy; 17) Respiratory System-Cardiovascular Diseases; 18) Solid Organ Transplant Surgery; 19) Transplant Nephrology and Dialysis; 20) Vascular Surgery.

**Table 3:** Risk of CDTB positivity in wards with CDTB positive analyses > 1 compared to wards with a single positive analysis in months with the highest frequency of positivity.

**Variable**		**Odds** **Ratio**	**95% CI**
**CDTB Positive** **Analyses** (number)	**Wards ** **Involved** (number)		
1	15	1	-
2	10	1.32	0.58-3.03
3	4	1.20	0.42-3.39
4	3	1.25	0.46-3.41
5	2	1.51	0.50-4.54
6	3	1.13	0.46-2.75
8	2	1.77	0.64-4.94
9	1	1.07	0.33-3.45
11	1	1.40	0.44-4.40

**Table 4:** Baseline characteristics of positive CDTB patients.

**Variable**	**Odds** **Ratio**	**95% CI**	**P-Value**
Extra-binomial variability due to ward	-	-	0.610
Sex			0.081
Females	Ref.	-	
Males	0.84	0.69-1.02	
Age, years			<0.001
0-64	Ref.	-	
65-79	1.43	1.10-1.87	
>=80	1.93	1.49-2.49	
Patients provenance			0.856
hospital	Ref.	-	
community	0.97	0.69-1.36	
Year of analysis			0.098
2010	Ref.	-	
2011	0.71	0.47-1.06	
2012	0.65	0.45-0.96	
2013	0.80	0.56-1.14	
2014	0.75	0.53-1.06	
2015	0.52	0.32-0.84	
Seasonality			0.029
SIN	1.15	1.01-1.32	
COS	1.13	0.99-1.28	

SIN, sine, COS, cosine, *i.e*., trigonometric components (see (Fig. **1**))

## LIST OF ABBREVIATIONS

CD: *Clostridium Difficile*
CDI: CD Infection
PCR: Polymerase Chain Reaction
CDTB: CD Toxin B
OR: Odds Ratio
CIR: Crude Incidence Rates
ISS: Istituto Superiore di Sanità
HCFA: Health-care Facility Associated
CO: Community Onset
CA: Community Associated
